# Lateral extra‐articular procedures in primary anterior cruciate ligament reconstruction reduce graft failure rates in elite and competitive athletes: A systematic review

**DOI:** 10.1002/jeo2.70908

**Published:** 2026-09-17

**Authors:** Laurin Kratochwil, Simon Kienberger, Adrian Deichsel, Tobias Gotterbarm, Philipp W. Winkler

**Affiliations:** ^1^ Medical University of Vienna Vienna Austria; ^2^ Department for Orthopedics and Traumatology Kepler University Hospital GmbH, Johannes Kepler University Linz Linz Austria; ^3^ Department for Trauma Hand and Reconstructive Surgery, University Hospital Muenster Muenster Germany

**Keywords:** anterior cruciate ligament reconstruction, athletes, knee instability, lateral extra‐articular tenodesis, orthopedic surgery

## Abstract

**Purpose:**

The aim of this systematic review was to evaluate clinical outcomes of combined primary anterior cruciate ligament reconstruction (ACLR) and lateral extra‐articular procedures (LEAP) in elite and competitive athletes. Where enough data were available, the review also aimed to differentiate between various LEAP techniques.

**Methods:**

A systematic search was conducted following the preferred reporting items for systematic reviews and meta‐analyses guidelines using the PubMed, Scopus, Embase and Web of Science databases. Two independent observers screened and assessed the studies; the data were analysed cumulatively where possible and reported narratively.

**Results:**

A total of 12 studies met the inclusion criteria, reporting data on 1.719 knees that underwent primary ACLR. An additional LEAP was performed in 783 cases, including anterolateral ligament reconstruction (ALLR), the modified Lemaire and MacIntosh Coker–Arnold techniques. Notably lower graft failure rates were observed, ranging from 0% to 6.5% in the combined ACLR and LEAP groups, compared to 0%–34.0% in the isolated ACLR groups. The cumulative graft failure rate was 3.4% (10/292) for combined ACLR and ALLR, and 2.5% (4/158) for combined ACLR and modified Lemaire, compared to 10.7% (81/758) for isolated ACLR. Return to sport (RTS) rates were similar, ranging from 85.7% to 100% in the combined ACLR and LEAP group, and from 85.6% to 100% in the isolated ACLR group.

**Conclusion:**

Based on this study, adding a LEAP to primary ACLR was associated with lower observed graft failure rates and improved knee stability, while return‐to‐sport rates remained similar in elite and competitive athletes.

**Level of Evidence:**

Level IV, systematic review.

## Graphical abstract

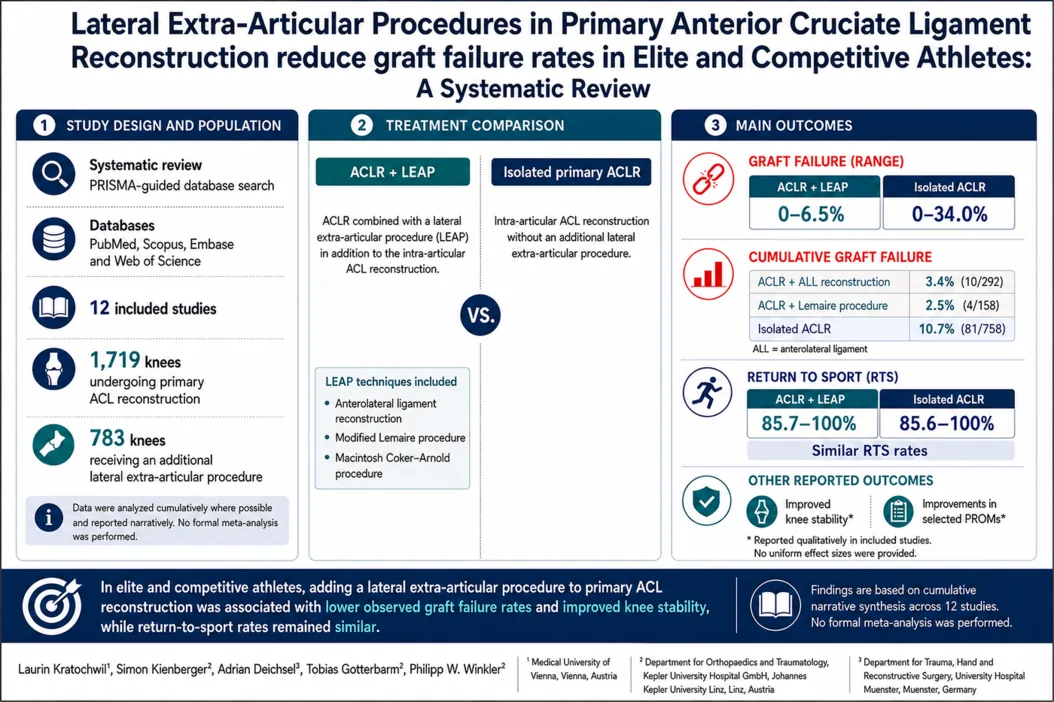

**Abbreviations**
ACL: anterior cruciate ligament
ACLR: anterior cruciate ligament reconstruction
ALL: anterolateral ligament
ALLR: anterolateral ligament reconstruction
ALRI: anterolateral rotatory knee instability
AP: anteroposterior
IKDC: International Knee Documentation Committee
LEAP: lateral extra‐articular procedure
LET: lateral extra‐articular tenodesis
PLT: peroneus longus tendon
PRISMA: preferred reporting items for systematic reviews and meta‐analyses
PROM: patient‐reported outcome measure
PROSPERO: International Prospective Register of Systematic Reviews
RTS: return to sport
SD: standard deviation

## INTRODUCTION

Anterior cruciate ligament (ACL) ruptures are among the most common sports injuries [3, 9, 60]. Professional football, handball, alpine skiing and other pivoting sports consistently report among the highest ACL injury incidences, with female athletes demonstrating an even greater injury risk than their male counterparts [3, 13, 39, 48]. Furthermore, evidence suggests that the incidence increases with the level of competition, with professional athletes facing a significantly higher risk than individuals in lower divisions [3, 13, 48]. ACL injuries result in athletes being absent from sport for months and require intensive functional rehabilitation [5, 19, 21, 22].

Most professional athletes ultimately return to sport (RTS) and regain their preinjury level of performance after anterior cruciate ligament reconstruction (ACLR) [18]. However, a relevant proportion of patients report persistent rotatory knee instability following ACLR, with anterolateral rotatory knee instability (ALRI) being a leading cause of ACLR failure [23, 25, 60]. The rate of secondary ACL injuries ranges from 6% to 34% [45, 57]. Young athletes in pivoting sports are particularly vulnerable, with up to a 15‐fold increased risk of ACL graft failure within the first year after ACLR [45].

Biomechanically, the anterolateral structures of the knee (including the iliotibial tract and anterolateral ligament [ALL]), synergistically resist excessive internal rotation and anterior translation of the tibia [1, 9, 14, 56]. In this context, the efficacy of lateral extra‐articular procedures (LEAP) has been recognised for over 50 years, since they were first performed as standalone interventions [25, 46, 47]. LEAP have shown to improve both subjective and objective stability, providing significant enhancements in clinical outcomes, when performed concomitantly to ACLR [42]. Consequently, they have experienced a resurgence of interest in the past decade, particularly in revision ACLR [6, 30, 32, 47]. More recently, indications for LEAP have expanded to include primary ACLR, supported by substantial scientific evidence and expert consensus statements [52, 60]. The additional procedure is justified by clinical results due to reduced revision rates, decreased laxity and rotatory instability and improved functional movement [9, 30, 53, 54].

Although several recent systematic reviews and meta‐analyses have evaluated LEAP in mixed populations, only a few have specifically focused on elite or competitive athletes, who represent a unique subgroup characterised by higher exposure and higher RTS demands [16].

The purpose of this systematic review was to evaluate the clinical outcomes of combined primary ACLR and LEAP in this patient cohort. In addition to assessing the overall effect of LEAP on ACL graft failure, a further objective was to explore whether the available evidence supports differences in graft failure rates between the various LEAP techniques. This technique‐specific analysis was intended to provide additional insight into the potential differential effects of the currently used procedures and thereby address an aspect that has received limited attention in previous systematic reviews. It was hypothesised that combined ACLR and LEAP would result in lower ACL graft failure rates, improved patient‐reported outcome measures (PROMs) and RTS rates compared to isolated ACLR. It was also hypothesised that there would be no difference in ACL graft failure rates between different LEAP techniques.

## METHODS

This systematic review was conducted following the preferred reporting items for systematic reviews and meta‐analyses (PRISMA) guidelines and is registered in the PROSPERO registry (CRD420251129990) [40, 41].

### Search strategy

A systematic search of the PubMed (MEDLINE), Scopus, Embase and Web of Science databases was carried out from August 2025 to October 2025. Two independent reviewers (K.L., K.S.) conducted the search and reviewed the included articles in detail to avoid possible risk of bias. Only papers published or translated in English were included. The search strategy can be found in Supplement S1. The research question was defined using the PICO strategy [50].

### Inclusion criteria

Studies involving athletes undergoing combined ACLR and LEAP met the inclusion criteria. For inclusion in this systematic review, the title or abstract, or the authors’ inclusion criteria had to mention key terms such as ‘elite’, ‘professional’, ‘high level of sport‘ or ‘competitive’. Studies with a level of evidence ranging from I to IV, both randomised and nonrandomised studies, case‐control studies, cohort studies and case series were considered.

Noncomparative case series were included because high‐quality comparative studies in elite athletes remain scarce. Excluding these studies would have substantially reduced the available evidence. Given the absence of a universally accepted definition of ‘elite athlete’ in literature, a previously published and widely accepted classification was adopted to ensure a transparent and reproducible study selection process.

According to the American Heart Association, an athlete is ‘someone who participates in organised team or individual sports that require regular competition against others and place a high value on excellence and achievement. This usually requires some form of systematic training’ [11]. The term ‘elite’ is often used to describe the standard of an athlete in literature [58]. However, the debate about how to define elite status is ongoing, making it difficult to select a truly homogeneous population [35]. Taking a quantitative approach, some studies define an elite athlete as someone who trains for more than 10 hours per week, whereas a professional athlete is someone who earns a living by practicing sports [36]. Other authors further distinguish between exercisers and athletes. Exercisers are individuals who participate in physical activity with the motivation to increase fitness, promote health, improve physique and learn or refine skills. On the other hand, athletes engage in physical activity with the primary goal of improving performance to bolster athletic excellence and/or achievement [36]. As there is still no consensus, the studies included in this systematic review feature patients, that could be considered as elite athletes as they were competing professionally, playing in high leagues or at a high level of sport (see Table 1) meeting the criteria of the American and European Heart Society that both emphasise ‘organised competition’ and an ‘award for excellence and success’ as integral components in their definition of an athlete [36]. Table 1 summarises the definitions and selection criteria used by the authors in their studies involving patients ranging from highly trained competitive individuals to professional athletes competing in different sports and at different levels, reflecting the heterogeneity of the currently available literature.

**Table 1:** Definitions of an athlete and additional eligibility criteria reported by the included studies.

First author, year of publication	Definition of Athlete/study population	Patient selection criteria		Sport
		Inclusion	Exclusion	
Hamido et al. 2020 [28]	‘High Level of sport’	combined ACL and ALL (MRI), Segond fracture, Pivot Shift III°, pivoting sports	history of knee surgery, ACL revision surgery or multiligamentous knee injury	Soccer, Handball, Crossfit, Basketball, Volleyball, Tennis, Kung‐fu, Fencing
Goncharov et al. 2019 [24]	‘professional’; participation in competitions	Age 16–40 years	Previous surgeries on the affected knee joint	n.r.
Arora et al. 2024 [2]	‘patients actively playing […] at either the state, national or international level’	Age > 18 years	Local level players, multi‐ligament injuries, previous history of ACL surgery	Kabbadi
Rosenstiel et al. 2019 [49]	(SANTI Group) Participating in competition at national and/or international levels at the time of injury		Age <15 years at the time of surgery, injury to the collateral ligaments of severity > grade 2, and any other ligament injury	Football, Rugby, Basket, Handball, Skiing, Hockey, Motocross
Balendra et al. 2022 [4]	‘Professional’	Age > 17 years	Surgical treatment of current or previous ligamentous knee injury	Football
Parmar et al. 2025 [44]	High school and collegiate players were required to be playing competitively		High school and collegiate athletes participating in recreational or intramural sports	n.r.
Guy et al. 2022 [26]	French national ski team database		Additional major surgical procedure (e.g., multiligament reconstruction) or history of ACL injury	Skiing
Shanthini et al. 2025 [20]	State or national level leagues	Pivot Shift III° Age 20–30 years	Multiligament knee injury	Football
Borque et al. 2025 [7]	‘Professional […] players’		Multiligament knee injury, Revision ACLR	Rugby
Borque et al. 2022 [8]	One who is paid to perform one's sport or one who participates in national‐ or international‐level competitions in amateur sports	Age > 15	Revision ACLR, multiligament surgery, patients not receiving an autograft hamstring or a patellar tendon graft	Soccer (56%) Rugby (30%) Cricket, Field Hockey, Judo, Gymnastics, Netball
Guzzini et al. 2016 [27]	High preinjury level (national team, first or second division)		Previous surgical procedures on the same knee, multi‐ligamentous injuries	Football (FIGC) for elite female players
Hopper et al. 2022 [31]	(SANTI Group) ‘professional athletes’		Major concomitant procedures	n.r.

Abbreviations: ACLR, anterior cruciate ligament (reconstruction); ALL, anterolateral ligament; FIGC, Federazione Italiana Giuoco Calcio; MRI, Magnetic Resonance Imaging; n.r., not reported.

Furthermore, a minimum follow‐up period of 12 months was required, as were descriptions and reports of clinical outcomes, including at least one of the following: ACL graft failure rate, RTS rates, RTS level, validated PROMs and knee laxity. Information on the indication and the surgical technique for the LEAP was collected.

### Exclusion criteria

The exclusion criteria were as follows: case reports, studies reporting purely radiological outcomes, and biomechanical, animal or cadaveric studies. Further exclusion criteria were revision ACLR and studies that included noncompetitive athletes, or patients who were not clearly identified as such, as well as skeletally immature patients or adolescents <15 years at the time of ACLR, as this was determined by the study selection. One of the included studies had a lower age limit. This is also shown in Table 1.

### Data selection and collection process

First, the search results were screened by title. As soon as potentially relevant studies were found, the abstract was read. Irrelevant studies were then excluded. Only those from the abstract screening were considered for full‐text screening and assessed for eligibility. Finally, the reference lists of all the studies included after the database search were screened by title and, if necessary, by abstract to ensure that no relevant publications were overlooked. Data were then extracted and processed using Microsoft Excel (version 16.103.1).

All records were screened independently by two reviewers (K.L., K.S.). Any disagreements were resolved through discussion with a third reviewer (W.P.W.). Data extraction was performed independently by two reviewers; discrepancies were solved in a similar manner as in the screening process.

For each article, the following characteristics were collected: first author, year of publication, sample size, age, sex distribution, concomitant injuries, time to surgery, follow‐up, surgical protocol, indication for LEAP, LEAP technique, graft failure rates, knee laxity, Lachman Test, Pivot Shift Test, Lysholm Score, Tegner Activity Scale, International Knee Documentation Committee (IKDC), RTS, RTS level, RTS timepoint.

Graft failure rates were considered as primary outcomes, and the other clinical scores and measurements and RTS metrics as secondary outcomes.

### Risk of bias assessment

Methodological quality was assessed according to study design. The randomised controlled trial was evaluated using the Cochrane Risk of Bias 2 (RoB2) tool, whereas nonrandomised comparative and noncomparative studies were assessed using the methodological index for nonrandomised studies (MINORS) [51]. This index includes a rating system comprising 12 subitems, each worth two points (0 [not reported], 1 [reported but inadequate] or 2 [reported and adequate]), four of these can only be applied to comparative studies. It results in a maximum score of 24 for comparative studies and 16 for noncomparative studies, evaluating criteria such as the clarity of the study aim, inclusion of consecutive patients, prospective data collection, appropriateness of endpoints, unbiased assessment of endpoints, adequacy of follow‐up duration, loss to follow‐up, prospective calculation of study size, presence of an adequate control group, contemporaneous management of groups, baseline equivalence, and appropriateness of statistical analyses. A figure showing the quality evaluation process using the Cochrane RoB2 and MINORS can be found in Supplement S2.

### Data analysis and synthesis

Due to heterogeneity in the study design and outcomes, a meta‐analysis was not feasible. No statistical analysis was performed. Instead, the results were reported narratively. When one study featured several nonathletes, only the data regarding and concerning the athlete‐cohort was described and recorded. Whenever there was sufficient data, the cumulative values of the clinical outcomes for the ACLR + LEAP group were analysed to identify patterns and differences of the various LEAP techniques.

### Declaration of generative AI and AI‐assisted technologies in the writing process

During the preparation of this work, the authors used ‘Deepl Write’ to check grammar and improve readability. Having used this tool, the authors reviewed and edited the content as required, taking full responsibility for its publication.

## RESULTS

### Literature search results

**Figure 1:**
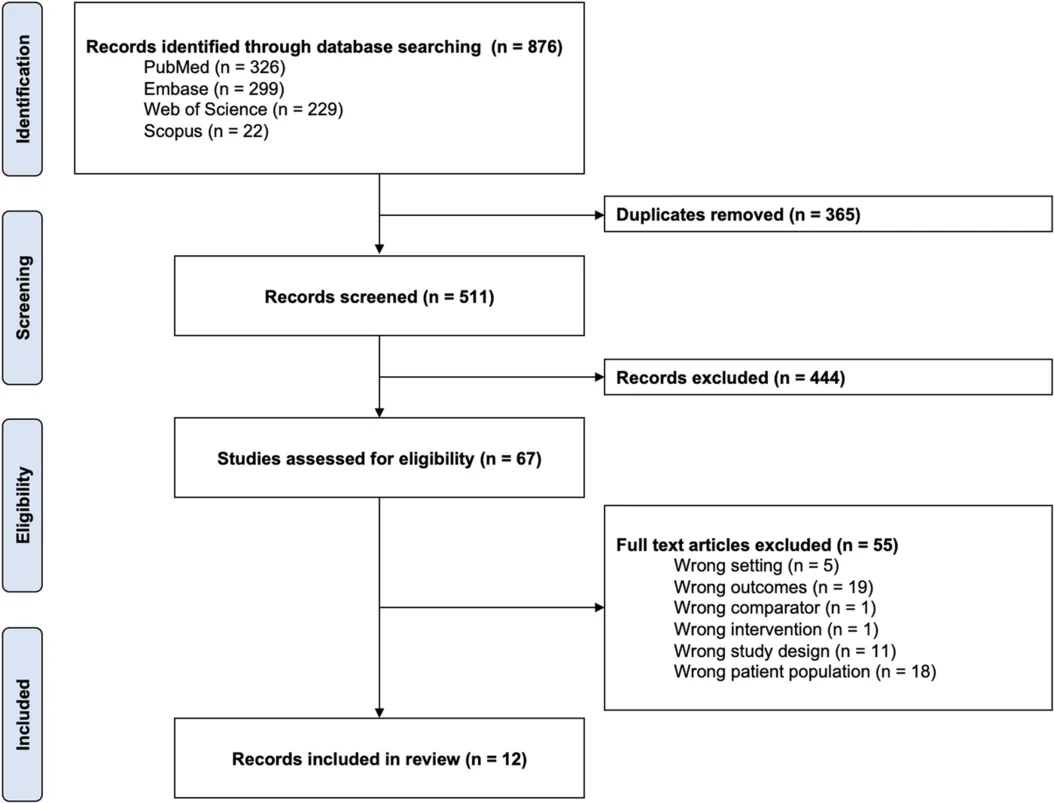
Flowchart showing the literature selection process.

The initial search of the databases yielded 876 records. After removing duplicates (*n* = 365), another 444 studies were excluded after screening the titles and abstracts. Eventually, 67 were assessed for eligibility. Ultimately, both reviewers included 12 records in the final analysis after evaluating full texts. The PRISMA diagram is shown in Figure 1.

### Demographics

All relevant patient and study characteristics are reported in Table 2. A total of 1.719 knees were included. In 783 of those cases, a combined primary ACLR with a LEAP was performed. Four studies did not have a control group (isolated ACLR, see Table 2). Only one Level I study met the inclusion criteria.

**Table 2:** Characteristics of the included studies.

First author, year of publication	LoE	RoB2/MINORS score	Sample size included in analysis, *n*			Sex male/female, *n*			Age, years, mean ± SD (range)			Median or mean follow up ± SD (range) [months]		Time to surgery ± SD (range) [months]; n (%)	
			Overall	Isolated ACLR	ACLR + LEAP	Overall	Isolated ACLR	ACLR + LEAP	Overall	Isolated ACLR	ACLR + LEAP	Isolated ACLR	ACLR + LEAP	Isolated ACLR	ACLR + LEAP
Hamido et al. 2020 [28]	I	‘Some concerns’	102	52	50	102/0	52/0	50/0	n.a.	26 (18–40)	24 (18–33)	60 (55–65)	60 (55–65)	3 (2.5–3.7)	3 (2.5–3.8)
Goncharov et al. 2019 [24]	II	19	48	30	18	n.a.	n.a.	n.a.	n.a.	n.a.	n.a.	24	24	n.a.	n.a.
Arora et al. 2024 [2]	II	14	93	‐	93	78/15	‐	78/15	22.4 ± 2.7	‐	22.4 ± 2.7	‐	12	‐	n.a.
Rosenstiel et al. 2019 [49]	IV	11	70	‐	70	48/22	‐	48/22	23.2 (15–37)	‐	23.2 (15–37)	‐	3.9* (2–7)	‐	1.1 (0.1–7)
Balendra et al. 2022 [4]	IV	18	232	178	54	205/27	n.a.	n.a.	23.3 ± 4.4	n.a.	n.a.	n.a.	n.a.	<14 days: 106 (45.7) 15–28 days: 78 (33.6) >28 days: 45 (19.4)^a^	
Parmar et al. 2025 [44]	III	17	133	90	43	0/133	0/90	0/43	n.a.	17.8 ± 2.4	18.2 ± 3.1	36.1 ± 12.0	39.0 ± 14.0	n.a.	n.a.
Guy et al. 2022 [26]	III	17	81	50	31	45/36	25/25	20/11	22.5 ± 4.5	21.0 ± 3.9 (13 – 32)	24.9 ± 4.5 (19 – 36)	24	24	n.a.	n.a.
Shanthini et al. 2025 [20]	II	11	22	‐	22	22/0	‐	22/0	22.4 ± 3.1	‐	22.4 ± 3.1	‐	12	‐	n.a.
Borque et al. 2025 [7]	III	16	125	88	37	114/11	n.a.	n.a.	23.4 ± 4.0	n.a.	n.a.	3.7* (2–12)		<14 days: 64 (51) 15–28 days: 39 (31) >28 days: 22 (18)^a^	
Borque et al. 2022 [8]	III	18	455	338	117	376/79	274/64	102/15	22.5 ± 4.7	22.9 ± 4.9	21.5 ± 4.1	>24		n.a.	n.a.
Guzzini et al. 2016 [27]	II	12	16	‐	16	0/16	‐	0/16	26.4 ± 4.7	‐	n.a.	‐	72.6 ± 8.1	‐	4.5 ± 2.5**
Hopper et al. 2022 [31]	III	20	342	110	232	274/68	n.a.	n.a.	23.9 ± 5.2	n.a.	n.a.	140.8 ± 47.3	81.2 ± 41.8	2.0 ± 4.0^1^	

Abbreviations: ACLR, anterior cruciate ligament reconstruction; LEAP, lateral extra‐articular procedures; LoE, level of evidence; MINORS, methodological index for nonrandomised studies; *n*, number of patients; n.a., not available; RoB2, Cochrane Risk of Bias 2; SD, standard deviation.

*years.

**days.

^a^ reported data was not differentiated between isolated ACLR and combined ACLR and LEAP.

The included studies predominantly comprised male athletes. However, several noncomparative case series also included female athletes, although sex‐specific outcome analyses were not reported.

### Surgical technique and indications

This systematic review included studies in which ACLR was combined with three different LEAP techniques: anterolateral ligament reconstruction (ALLR), modified Lemaire tenodesis and the Arnold‐Coker modification of the MacIntosh tenodesis [12, 24, 55, 60].

Seven studies included patients undergoing ACLR combined with an additional ALLR [7, 8, 24, 26, 28, 31, 49], six studies used the modified Lemaire technique [2, 4, 20, 26, 31, 44], some of them performed both techniques (ALLR and modified Lemaire) and did not differentiate between them when reporting on clinical outcome measures. The Arnold Coker modification of the McIntosh procedure was described in one study [27]. The surgical protocol and details are reported in Table 3. The indications for LEAP were composed of joint‐specific factors (e.g., general ligamentous laxity (GLL), pivot shift grade 2 or 3, etc.) and patient‐specific factors (e.g., activity level, desire to return to high‐risk sports). Furthermore, the included studies revealed significant variations in the selection of grafts and concomitant procedures for ACLR in athletes. The hamstrings were most frequently used as an ACL graft.

**Table 3:** Surgical protocols and indications for lateral extra‐articular procedures in the included studies.

First author, year of publication	Indication for LEAP	LEAP	ACL	LEAP			Concomitant surgical procedures (*n*)	
			Graft choice		Flexion angle at fixation	Tibial rotation at fixation	Isolated ACLR	ACLR + LEAP
Hamido et al. 2020 [28]	Patients with a high‐grade pivot shift (III), Segond fracture, a high level of sports activity and those participating in sports involving frequent pivoting sports	ALLR	HS	GT	0°–15°	n.a.	mMR (2), mpME (8), lMR (2), lpME (4), mpME + lpME (1)	mMR (1), mpME (7), lMR (1), lpME (4)
Goncharov et al. 2019 [24]	n.a.	ALLR	BPTB	GT or ST	0°	Neutral	n.a.	n.a.
Arora et al. 2024 [2]	High‐demand and high‐risk populations such as young elite players with high‐grade ALRI	Lemaire	PLT	ITB	30°	Neutral	n.a.	n.a.
Rosenstiel et al. 2019 [49]	Professional athletes	ALLR	HS	GT	0°	Neutral	mMR (19), mpME (3), lMR (29), lpME (2)^a^	
Balendra et al. 2022 [4]	>7° hyperextension, GLL (Beighton > 4) varus or valgus malalignment > 7°, a posterior tibial slope of 12° or more, a ‘pivot‐glide’ in the normal knee, or history of ACL rupture on the contralateral limb	Lemaire	BPTB or HS	ITB	n.a.	n.a.	mMR (48), mpME (13), lMR (93), lpME (43)^a^	
Parmar et al. 2025 [44]	Greater‐grade pivot shift test or (GLL) based on Beighton score	Lemaire	BPTB, QT or HS	ITB	60°	n.a.	MR (47), pME (15)	MR (20), pME (8)
Guy et al. 2022 [26]	n.a.	ALLR or Lemaire	BPTB, QT or HS	ITB or GT	0°	n.a.	mMR (15), mpME (6), lMR (27), lpME (7)^a^	
Shanthini et al. 2025 [20]	Professional athletes with Pivot Shift III°	Lemaire	HS	ITB	30°	Neutral	mMR or mpME (6), lMR or lpME (5)^a^	
Borque et al. 2025 [7]	Elite athletes undergoing primary ACLR	ALLR	HS or BPTB	ITB	n.a.	n.a.	MR (64), pME (20)^a^	
Borque et al. 2022 [8]	Elite athletes undergoing primary ACLR	ALLR	HS or BPTB	ITB	n.a.	n.a.	MR (213), pME (80)^a^	
Guzzini et al. 2016 [27]	n.a.	MacIntosh modified Coker–Arnold	HS	ITB	‘Knee flexed’	Max. external rotation	mpME (4), lpME (1), mpME + lpME (1)^a^	
Hopper et al. 2022 [31]	Based on patient characteristics, patient choice and evolving indications for concomitant LEAP noted by the senior surgeon	ALLR or Lemaire	BPTB, QT or HS	ITB	0°	Neutral	n.a.	n.a.

Abbreviations: ACLR, anterior cruciate ligament (reconstruction); ALLR, anterolateral ligament reconstruction; ALRI, anterolateral rotatory knee instability; BPTB, bone‐patellar tendon‐bone graft; GLL, generalised ligamentous laxity; GT, gracilis tendon; HS, Hamstrings; lMR, lateral meniscus repair; lpME, lateral partial meniscectomy; ITB, iliotibial band; LEAP, lateral extra‐articular procedure; mMR, medial meniscus repair; mpME, medial partial meniscectomy; MR, meniscal repair; n, number of patients; n.a., not available; PLT, peroneus longus tendon; pME, partial meniscectomy; ST, semitendinosus tendon; QT, quadriceps tendon.

^a^ Reported data were not differentiated between isolated ACLR and combined ACLR and LEAP.

Ten studies included patients who received a hamstring graft; among these studies, six also included BPTB grafts, and three included quadriceps tendon (QT) grafts in the analysis. In one study, patients received a Peroneus longus (PLT) autograft [2]. There was no study using allografts. Overall, there were few studies in which the entire patient cohort received the same graft: four studies with exclusively hamstring grafts [20, 27, 28, 49], one using BTPB [25] only and one with a PLT graft [2], as mentioned above.

### Clinical outcomes and PROMs

Table 4 shows the clinical and patient‐related outcomes that were measured or collected before and after surgery, including effect measures (Standard deviation, range, percentage), reflecting the results of the respective last follow‐up assessment. In the randomised controlled trial by Hamido et al. [28], five patients (9.6%) in the group that underwent isolated ACLR demonstrated an anterior tibial translation (ATT) of more than 5 mm postoperatively. In contrast, none of the patients in the group of combined ACLR + LEAP showed an ATT > 5 mm; this difference was statistically significant (*p* < 0.001). Four (4.2%) patients in Arora et al.'s [2] study, that underwent combined ACLR + LEAP (modified Lemaire) reported postoperatively about subjective laxity. Guzzini et al. [27] reported that 11 patients (67%) exhibited preoperative ATT exceeding 5 mm. After combined ACLR and Arnold Coker modified McIntosh procedure, none of the patients showed laxity above this threshold.

**Table 4:** Clinical outcomes reported in the included studies.

First author, year of publication	Surgical procedure	AP laxity ± SD [mm] (range)		*p*‐value	Negative Lachman, *n* (%);	*p*‐value	Grade 0 pivot shift, *n* (%)	*p*‐value	Lysholm ± SD (range)		*p*‐value	Tegner Level or Score ± SD (range); Level, *n* (%)		*P*‐value	Subjective IKDC ± SD (range) objective IKDC, grade, *n* (%)		*p*‐value
		**⇐**	**⇒**		**⇒**		**⇒**		**⇐**	**⇒**		**⇐**	**⇒**		**⇐**	**⇒**	
Hamido et al. 2020 [28]	Isolated ACLR	n.a.	2.5 ± 0.7 (1.2–6.1)	**<0.001**	44 (84.6)		43 (82.6)	< **0.001**	74 ± 14.5 (40– 82)	94 ± 4.5 (65–100)		6.9 ± 1.6 (5.0–8.0)	7.8 ± 1.4 (6.0– 9.0)		Grade A: 0	Grade A: 44 (84.6)	**<0.001**
	ALLR	n.a.	1.2 ± 0.7 (1.2–3.2)		48 (96.0)		48 (96.0)		72 ± 13.5 (40– 83)	96 ± 5.0 (70–100)		6.4 ± 1.2 (5.0–9.0)	7.9 ± 0.8 (5.0– 9.0)		Grade A: 0	Grade A: 48 (96.0)	
Goncharov et al. 2019 [24]	Isolated ACLR	n.a.	<3 mm: 17 (56.7)		n.a.		19 (63.3)		n.a.	n.a.		69.6 ± 3.5	92.1 ± 3.9	*<0.01*	73.4 ± 3.2	90.3 ± 3.7	*<0.01*
	ALLR	n.a.	<3 mm: 18 (100)		n.a.		18 (100)		n.a.	n.a.		72.6 ± 6.5	97.4 ± 1.2		63.1 ± 4.8	96.3 ± 1.8	
Arora et al. 2024 [2]	Lemaire	n.a.	n.a.		n.a.		n.a.		78.5	100.0		n.a.	n.a.		68.0	87.0	
Rosenstiel et al. 2019 [49]	ALLR	7.1 ± 1.4	0.4 ± 0.9	*<0.0001*	n.a.		66 (94.3)	*<0.0001*	48.4 ± 12.5	94.4 ± 7.5	*<0.0001*	9.3 ± 1*	8.8 ± 1.5	*0.0004*	56.1 ± 13.3Grade A: 0 (0)Grade D: 31 (44.3)	90.5 ± 7.6 Grade A: 65 (92.9) Grade D: 0 (0)	*<0.0001*
Balendra et al. 2022 [4]	Isolated ACLR	n.a.	n.a.		n.a.		n.a.		n.a.	n.a.		Level 9: 147 (63.4)* Level 10: 85 (36.6)*	n.a.		n.a.	n.a.	
	Lemaire																
Parmar et al. 2025 [44]	Isolated ACLR	n.a.	n.a.		n.a.		65 (72.2)	**0.028**	50.4 ± 10.2	91.2 ± 5.5	**0.823**	n.a.	n.a.		51.2 ± 11.8	88.3 ± 6.9	**.012**
	Lemaire	n.a.	n.a.		n.a.		39 (90.7)		49.8 ± 8.7	90.9 ± 6.2		n.a.	n.a.		51.2 ± 6.7	91.3 ± 5.1	
Guy et al. 2022 [26]	Isolated ACLR	6.3 ± 1.4 (3–10)	n.a.		n.a.		n.a.		n.a.	n.a.		n.a.	n.a.		n.a.	n.a.	
	ALLR or Lemaire	6.5 ± 1.6 (5–10)	n.a.		n.a.		n.a.		n.a.	n.a.		n.a.	n.a.		n.a.	n.a.	
Shanthini et al. 2025 [20]	Lemaire	n.a.	n.a.		n.a.		n.a.		n.a.	n.a.		46.7 ± 10.6	89.4 ± 5.7	*<0.0001*	48.2 ± 12.4	86.2 ± 6.8	*<0.0001*
Borque et al. 2025 [7]	Isolated ACLR	n.a.	n.a.		n.a.		n.a.		n.a.	n.a.		n.a.	n.a.		n.a.	n.a.	
	ALLR	n.a.	n.a.		n.a.		n.a.		n.a.	n.a.		n.a.	n.a.		n.a.	n.a.	
Borque et al. 2022 [8]	Isolated ACLR	n.a.	n.a.		n.a.		n.a.		n.a.	n.a.		n.a.	n.a.		n.a.	n.a.	
	ALLR	n.a.	n.a.		n.a.		n.a.		n.a.	n.a.		n.a.	n.a.		n.a.	n.a.	
Guzzini et al. 2016 [27]	MacIntosh modified Coker–Arnold	10.2 ± 0.77 (9–11)	2.2 ± 0.7 (1–3)	*<0.05*	16 (100)	*<0.05*	14 (87.5)	*<0.05*	52.5 ± 5.1	91 ± 2.3	*<0.05*	8.1 ± 1.1	7.1 ± 1.9	*<0.05*	n.a.	87.1 ± 13	*<0.05*
Hopper et al. 2022 [31]	Isolated ACLR	6.8 ± 1.9	0.3 (0–2)		n.a.		n.a.		n.a.	n.a.		n.a.	n.a.		n.a.	n.a.	
	ALLR or Lemaire				n.a.		n.a.		n.a.	n.a.		n.a.	n.a.		n.a.	n.a.	

Abbreviations: **⇐** before surgery; **⇒** after surgery; ACLR, anterior cruciate ligament reconstruction; ALLR, anterolateral ligament reconstruction; AP, anteroposterior; IKDC, International Knee Documentation Commitee; LEAP, lateral extra‐articular procedure; n, number of patients; n.a., not available; SD, standard deviation.

*preinjury value; bold: difference between combined ACLR and LEAP versus isolated ACLR; *cursive: difference compared to preoperative values.*

In Hamido et al.'s study [28], combined ACLR + LEAP showed significantly lower joint AP laxity than isolated ACLR (2.5 ± 0.7 mm [1.2–6.1] vs. 1.2 ± 0.7 mm [1.2–3.2]). Rosenstiel et al. [49] and Guzzini et al. [27] demonstrated significant improvements of AP laxity compared to preoperative values. Two studies [28, 44] demonstrated a significant difference in the number of patients with a postoperative grade 0 Pivot Shift between isolated ACLR and combined ACLR + LEAP, favouring the combined ACLR + LEAP group. The same two studies also demonstrated significant improvements in the IKDC when ACLR was combined with LEAP, compared to isolated ACLR. Five studies [4, 7, 8, 26, 31] did not analyze any PROMs, but reported graft failure rates as an outcome.

### Graft failure

As shown in Table 5, graft failure rates seemed to be remarkedly lower by performing combined ACLR + LEAP, ranging from 0% to 6.5% in the combined ACLR and LEAP population and 0% to 34% in the isolated ACLR group. Five of the comparative studies showed reduced graft failure rates. Three of these studies observed a statistically significant difference [8, 26, 31].

**Table 5:** Graft failure rates following anterior cruciate ligament reconstruction with or without lateral extra‐articular procedures in the included studies.

Author, year of publication	LEAP	Isolated ACLR, *n* (%)	ACLR + LEAP, *n* (%)	*p*‐value	Multivariable analysis
Hamido et al. 2020 [28]	ALLR	5 (9.6)	0		
Goncharov et al. 2019 [24]	ALLR	0	0		
Arora et al. 2024 [2]	Lemaire	‐	2 (2.1)		
Rosenstiel et al. 2019 [49]	ALLR	‐	4 (5.7)		
Balendra et al. 2022 [4]	Lemaire	12 (5.2)^a^			
Parmar et al. 2025 [44]	Lemaire	3 (3.3)	2 (4.7)	0.658	
Guy et al. 2022 [26]	ALLR or Lemaire	17 (34.0)	2 (6.5)	**0.0060**	**0.0412**
Shanthini et al. 2025 [20]	Lemaire	‐	0 (0)		
Borque et al. 2025 [7]	ALLR	7 (8.0)	2 (5.5)	0.615	
Borque et al. 2022 [8]	ALLR	32 (9.5)	4 (3.4)	**0.045**	**0.029**
Guzzini et al. 2016 [27]	MacIntosh modified Coker–Arnold	‐	0 (0)		
Hopper et al. 2022 [31]	ALLR or Lemaire	17 (15.5)	14 (6.0)	**0.0105**	**0.0164**

Abbreviations: ACLR, anterior cruciate ligament reconstruction; ALLR, anterolateral ligament reconstruction; LEAP, lateral extra‐articular procedure; *n*, number of patients; ‐: missing control group.

^a^ The reported data were not differentiated between isolated ACLR and combined ACLR and LEAP.

Across five studies reporting ACL graft failure following combined ACLR + ALLR [7, 8, 24, 28, 49], the cumulative failure rate was 3.4% (10/292). In four of these studies, including a control group with patients undergoing isolated ACLR, the cumulative failure rate in the control group was 8.7% (44/508). In three studies combining ACLR with a Lemaire tenodesis [2, 20, 44], resulted in a cumulative ACL graft failure rate of 2.5% (4/158).

Taking into account further studies that employed both LEAP techniques without distinguishing between them when reporting graft failure rates [7, 8, 24, 26, 28, 31, 44], the cumulative ACL graft failure rate in the ACLR + ALLR or Lemaire group was 4,5% (24/528) and 10,7% (81/758) in the isolated ACLR population, suggesting a numerically lower graft failure rate following combined procedures.

### RTS

RTS rates between the ACLR + LEAP population and those undergoing isolated ACLR were high in both groups, with rates over 85% throughout all studies, ranging from 85.7% to 100% in the combined ACLR + LEAP group. Balendra [4] and Parmar et al. [44] showed that athletes undergoing combined ACLR + LEAP had a higher RTS rate and an earlier RTS compared to isolated ACLR, although the difference was not statistically significant, respectively 96.3% versus 96.0% and 90.7% versus 85.6%. Balendra et al. [4] defined RTP as playing at least one competitive football match at a professional level following ACLR. Goncharov et al. [24] showed a difference in RTS level between professional athletes that had isolated ACLR and professional athletes that had combined ACLR + ALLR, where 100% could return to a competitive level of sport, whereas only 50% in the isolated ACLR group regained that level of sport.

The average RTS time point was notably earlier after 8.9 ± 1.4 [44] and 10.4 ± 3.8 [4] months compared to 9.4 ± 2.5 and 11.1 ± 3.0 months in the isolated ACLR group. Details are reported in Table 6.

**Table 6:** Return to sport outcomes reported in the included studies.

Author, year of publication	LEAP	RTS rate, *n* (%)		RTS level, same or higher, *n* (%)			RTS time point [months] (range)		Career length, [years ± SD] Playing professionally > 5 years (%)		
		Isolated ACLR	ACLR + LEAP	Isolated ACLR		ACLR + LEAP	Isolated ACLR	ACLR + LEAP	Isolated ACLR		ACLR + LEAP
Hamido et al. 2020 [28]	ALLR	52 (100)^a^		n.a.		n.a.	n.a.		n.a.		
Goncharov et al. 2019 [24]	ALLR	n.a.	n.a.	5 (50)*		10 (100)*	n.a.		n.a.		
Arora et al. 2024 [2]	Lemaire	‐	92 (98.9)	‐		80 (86)	‐	7.8	n.a.		
Rosenstiel et al. 2019 [49]	ALLR	‐	60 (85.7)	‐		60 (85.7)	‐	7.9 (5–12)	n.a.		
Balendra et al. 2022 [4]	Lemaire	170 (96.0)	52 (96.3)	209 (90.1)^a^			11.1 ± 3.0	10.4 ± 3.8	n.a.		
Parmar et al. 2025 [44]	Lemaire	77 (85.6)	39 (90.7)	69 (76.7)		34 (79.1)	9.4 ± 2.5	8.9 ± 1.4	n.a.		
Guy et al. 2022 [26]	ALLR or Lemaire	50 (100)	31 (100)	n.a.			n.a.		4.0 ± 3.4**		2.5 ± 2.3**
Shanthini et al. 2025 [20]	Lemaire	‐	22 (100)	‐		18 (82)	n.a.		n.a.		
Borque et al. 2025 [7]	ALLR	115 (92)^a^		110 (88)^a^			9.6^a^		5.9 ± 3.4^a^		
									70%		88%
Borque et al. 2022 [8]	ALLR	400 (88)^a^					10.1 ± 3.1^a^		n.a.		
Guzzini et al. 2016 [27]	MacIntosh modified Coker–Arnold	‐	16 (100)	‐	16 (100)		n.a.		n.a.		
Hopper et al. 2022 [31]	ALLR or Lemaire	324 (94.7)^a^		n.a.			n.a.		n.a.		

Abbreviations: ACLR, anterior cruciate ligament reconstruction; ALLR, anterolateral ligament reconstruction; LEAP, lateral extra‐articular procedure; RTS, return to sport; n, number of patients; n.a., not available; SD, standard deviation; ‐, missing control group.

* Only professional athletes are reported in the table.

** Exposure time defined as the duration that an ACL graft was subjected to the demands of elite skiing and therefore at high risk of rupture.

^a^ The reported data were not differentiated between isolated ACLR and combined ACLR and LEAP.

Only one study [7] evaluated career longevity following ACLR + LEAP and reported prolonged professional career participation in the combined group, with 88% playing professionally after 5 years after receiving a combined ACLR + LEAP compared to 70% with an isolated ACLR.

## DISCUSSION

The most important finding of this systematic review was the notably lower graft failure rate observed in elite and competitive athletes undergoing combined ACLR + LEAP compared to isolated ACLR.

A recent meta‐analysis by D'Ambrosi et al. [16] reported similar findings, including a reduced rerupture rate and a reduced risk of rerupture in elite athletes. The present review expands upon this evidence by including a broader range of eligible studies and providing a more detailed critical appraisal of the current literature, including methodological limitations, sources of heterogeneity and implications for clinical decision‐making. In addition, the present review specifically explored whether differences between the various LEAP techniques may influence graft failure. This technique‐specific aspect represents an additional clinical question that has received limited attention in previous systematic reviews.

### LEAP technique

The available evidence does not currently allow a clear distinction between individual LEAP techniques with regard to ACL graft protection.

Helito et al. [30] were among the first to directly compare different LEAP techniques. No clinically relevant differences between ALLR and lateral extra‐articular tenodesis (LET) were identified with regard to graft failure, postoperative pivot shift or laxity in the revision setting. The only observed differences concerned postoperative Lysholm scores and lateral knee pain, with higher Lysholm scores (89.0 ± 10.2 vs. 86.9 ± 6.9) and a shorter duration of lateral knee pain reported following ALLR (2.2 ± 1.3 vs. 4.3 ± 2.1 months). Although these findings were obtained in patients undergoing revision ACLR, they provide some support for the biomechanical rationale of lateral extra‐articular augmentation. However, extrapolation to primary ACLR should be performed with caution.

Guy et al. [26], who reported a reduction in ACL graft failure rates of almost 30% after combined ACLR + LEAP versus isolated ACLR, included both ALLR and the Lemaire technique in their study. While the rate of 6.5% in their analysis is low, it remains comparatively high, as other studies in this review, that differentiated between the techniques, report rates of between 0% and 4.7% for ACLR + Lemaire tenodesis and between 0% and 5.7% for ACLR + ALLR.

Overall, the limited number of direct comparative studies and the heterogeneity of the available evidence preclude a definitive conclusion regarding the superiority of a specific LEAP technique. Further high‐quality head‐to‐head studies are required to determine whether the choice of lateral augmentation technique influences graft failure, rotational stability, postoperative symptoms or other clinically relevant outcomes.

Concerning surgical technique, the reported flexion angles for LEAP (graft) fixation ranged from full extension to 60° of knee flexion. Evidence shows that LET combined with ACLR can effectively restore stability across various flexion angles [15, 34].

### Graft selection

Graft selection represents another potentially important factor when interpreting the observed reduction in graft failure. Borque et al. [8] demonstrated in a multivariate logistic regression analysis that the addition of LEAP to primary ACLR remained associated with a reduced risk of graft failure after accounting for age and sex. In a second analysis, no significant difference in graft failure was observed between patellar tendon and hamstring tendon grafts when combined with ALLR. Nevertheless, the variation in intraarticular graft selection across the included studies represents a potential source of heterogeneity. Experimental and clinical studies have suggested that the additional restraint provided by a LEAP may be particularly beneficial when combined with hamstring or peroneus tendon autografts compared to other graft types [17]. As hamstring tendon autografts predominated in the included studies, the favourable outcomes observed in the present review may partially reflect this graft distribution. However, the limited number of studies using alternative graft types, along with inconsistent reporting and additional potential confounding factors such as concomitant procedures, seasonal timing of injury or rehabilitation protocols, meant that meaningful subgroup analyses could not be performed and the results could only be reported narratively.

### Sex

Sex distribution also varied across the included studies, with several noncomparative case series including both male and female athletes without reporting sex‐specific outcomes. As sex can affect ACL injury mechanisms, graft failure risk and RTS outcomes, this represents another potential source of clinical heterogeneity that could not be accounted for in the present review. It must be noted that female athletes tend to end their careers earlier following ACL injury, experience longer periods of absence of sport and have a substantially higher risk of graft failure [5]. They are also less likely to RTS compared to male athletes [10]. Female athletes are therefore particularly vulnerable within this already high‐risk group and require gender‐specific recommendations, individualised indications and robust evidence to guide sustainable, sex‐specific medical care.

### RTS

Recent evidence shows that patients receiving a LEAP in addition to ACLR return to higher postoperative activity levels and are more likely to return to their pre‐injury level of sport [33]. This systematic review supports these findings, as 79,1–100% of athletes reached their pre‐injury level when a LEAP was added to primary ACLR, whereas other studies report RTP to the same level rates of 58%–73.1% [37, 38, 43].

The available evidence regarding the timing of RTS is less consistent.

Although current rehabilitation concepts increasingly favour criteria‐based progression [29], which may result in longer rehabilitation than time‐based protocols [9], comparative studies by Balendra [4] and Parmar et al. [44] showed that athletes undergoing ACLR + LEAP returned earlier. The difference was not statistically significant. Because RTS timing was not consistently reported, no firm conclusions can be drawn. An earlier return should not be interpreted as an indication for LEAP, as rehabilitation progression should remain guided by objective clinical and functional criteria [59].

For elite and competitive athletes, RTS is crucial, but it may not fully capture successful recovery. Return to pre‐injury performance level, sustained participation in professional competition, and career longevity may represent more meaningful outcomes. However, these outcomes were reported inconsistently across the included studies, preventing a systematic synthesis. Only Guy et al. [26] and Borque et al. [7] reported differences in professional participation following combined ACLR and LEAP. 88% of players who underwent ACLR + ALLR were still competing at a professional level 5 years postoperatively, compared to nearly 20% fewer in the isolated ACLR group.

Time at risk should also be considered when interpreting graft failure. A binary assessment of graft failure does not account for the duration during which an athlete remained exposed to competitive sport before failure. Hopper et al. [31] demonstrated a significant difference in ACL graft survivorship between isolated ACLR and ACLR + LEAP (*p* = *0.0105*). Balendra et al. [4] reported time to graft failure, with 5.2% of athletes sustaining a graft rupture within the first postoperative year, including five failures occurring before return to competitive sport. Such analyses may provide a more clinically meaningful assessment of graft survival in elite athletes. Future studies should therefore report time‐to‐failure and exposure time in addition to cumulative graft failure rates.

An illustrative finding was reported by Arora et al. [2], who described a patient who continued to compete at an elite level in kabaddi despite complete ACL graft failure, with an intact LET. Although this observation cannot be used to establish a causal relationship, it highlights the potential importance of the lateral structures in maintaining rotational stability and athletic function following ACL graft failure.

### Sport‐specific considerations

As elite athletes participating in alpine skiing, rugby, soccer, football and other pivoting sports have substantially different movement patterns, biomechanical demands, exposure and RTS criteria, any pooled interpretation should be treated with caution. Therefore, the present findings should not be interpreted as evidence that the benefit associated with LEAP is of the same magnitude across all sports. Due to the limited number of available studies within individual sports, sport‐specific subgroup analyses were not feasible and should be addressed in future research.

Similarly, although an established definition of an elite athlete was applied where possible, the included studies represented different levels of athletic participation, ranging from professional athletes to highly trained competitive athletes. The available evidence, therefore, supports the consideration of LEAP in high‐risk athletic populations but does not establish whether the observed effect is uniform across individual sports or levels of competition.

Moreover, the overall level of evidence was limited. Only one randomised controlled trial was identified, and the remaining studies predominantly consisted of Level III and IV evidence, including several noncomparative case series. While these studies provide valuable information regarding outcomes in elite athletes, they lack contemporaneous control groups and are inherently more susceptible to selection bias and confounding.

## CONCLUSION

This systematic review demonstrates the benefits of combining primary ACLR and LEAP in elite and competitive athletes, whose knees are subject to exceptionally high demands.

Adding a LEAP to primary ACLR was associated with lower observed graft failure rates and improved knee stability, while RTS rates remained similar. The limited reporting of Return to Performance and career longevity outcomes restricts conclusions regarding long‐term athletic success following the combined procedure. Furthermore, the available evidence remains insufficient to determine the optimal lateral extra‐articular technique.

## AUTHOR CONTRIBUTIONS

**Laurin Kratochwil**: Study design; literature screening; data extraction; study assessment; data analysis; statistical analysis. **Simon Kienberger**: Study design; literature screening; data extraction; study assessment; data analysis; statistical analysis; drafting manuscript, tables and figures. **Philipp W. Winkler**: Study assessment; data analysis; statistical analysis; drafting manuscript, tables and figures; revision manuscript, tables and figures. **Tobias Gotterbarm** and **Adrian Deichsel**: Revision manuscript, tables and figures. All authors contributed to the conception of the systematic review. The final version was approved by all authors.

## FUNDING INFORMATION

The authors have no funding to report.

## CONFLICT OF INTEREST STATEMENT

Adrian Deichsel is Web Editor for KSSTA. Philipp Winkler is Associate Editor for KSSTA. Member of the editorial board of JISAKOS. Tobias Gotterbarm: Grant and personal fees (Zimmer Biomet Europe, Depuye Synthes GmbH, Mathys AG), personal fees (Medacta, ImplanTec).

## ETHICS STATEMENT

The authors have nothing to report.

## Supporting information


Supplement 1.

Supplement 2.

## Data Availability

The data that support the findings of this study are available from the corresponding author upon reasonable request.
