# Supplementary material for: Lateral extra‐articular procedures in primary anterior cruciate ligament reconstruction reduce graft failure rates in elite and competitive athletes: A systematic review
Source: J Exp Orthop. 2026 Sep 17;13(3):e70908. doi: 10.1002/jeo2.70908 (PMC13585434; doi:10.1002/jeo2.70908)
Supplement: Supplementary file 1 [file JEO2-13-e70908-s001.pdf]

MINORS

	first author, year of publication	Borque et al. 2022	Goncharov et al. 2019	Arora et al. 2024	Rosenstiel et al. 2019	Shanthini et al. 2025	Guy et al. 2022	Balendra et al. 2022	Borque et al. 2025	Parmer et al. 2025	Guzzini et al. 2016	Hopper et al. 2022
1	A clearly stated aim	2	2	2	2	2	2	2	2	2	2	2
2	Inclusion of consecutive patients	2	2	2	2	2	2	2	2	2	2	2
3	Prospective collection of data	0	2	2	1	2	0	0	0	0	1	1
4	Endpoints appropriate to the aim of the study	2	2	2	2	2	2	2	2	2	2	2
5	Unbiased assessment of the study endpoint	1	1	1	0	0	0	0	0	0	1	1
6	Follow-up period appropriate to the aim of the study	2	2	1	2	1	2	2	2	2	2	2
7	Loss to follow up less than 5%	2	2	2	2	2	2	1	1	1	2	2
8	Prospective calculation of study size	0	0	2	0	0	0	2	0	0	0	0
	**Non-comparative studies**			**14**	**11**	**11**					**12**	
9	An adequate control group	2	1				2	2	2	2		2
10	Contemporary groups	2	2				2	2	2	2		2
11	Baseline equivalence of groups	1	1				1	1	1	2		2
12	Adequate statistical analyses	2	2				2	2	2	2		2
	**Comparative studies**	**18**	**19**				**17**	**18**	**16**	**17**		**20**

0 (not reported), 1 (reported but inadequate), or 2 (reported and adequate).

The maximum score for non-comparative studies is 16, and for comparative studies is 24

Cochrane RoB2

first author, year of publication	Overall RoB	comments
Hamido et al. 2020	Some Concerns	Randomization/Reporting
