# Supplementary material for: Lateral extra‐articular procedures in primary anterior cruciate ligament reconstruction reduce graft failure rates in elite and competitive athletes: A systematic review
Source: J Exp Orthop. 2026 Sep 17;13(3):e70908. doi: 10.1002/jeo2.70908 (PMC13585434; doi:10.1002/jeo2.70908)
Supplement: Supplementary file 2 [file JEO2-13-e70908-s002.pdf]

Supplement 1

Search Strategy ('anterior cruciate ligament reconstruction' OR 'acl reconstruction' OR aclr) AND ('lateral extra-articular*' OR 'lateral extraarticular*' OR 'anterolateral ligament reconstruction' OR 'anterolateral ligament*' OR allr OR lemaire OR ellison) AND (elite OR professional OR competitive OR athlete* OR 'return to sport*' OR 'return to play' OR rehabilitation)
